# An Essay on the Blood in Disease

**Published:** 1844-08

**Authors:** 


Art. VI.—*An Essay on the Blood in Disease.* By G. Andral, Professor of General Pathology and Therapeutics in the University of Paris. Translated from the French by J. F. Meigs, M.D., and Alfred Stillé, M.D. Philadelphia: Lea & Blanchard. 1844. pp. 126. 8vo.

Our readers require not to be informed that Andral occupies a conspicuous rank among the highest living medical authorities. His is not merely a European reputation: in all countries where medicine is pursued as a scientific profession his name is familiar, as one of the most philosophical, observant and practical physicians of the age. He has been now many years before the public as an author, and his analyses and researches are deemed among the most accurate and trustworthy, that have been given to the world on the subjects to which they relate. In the work before us he has taken up an inquiry which excited the deepest interest among medical men two centuries ago, but which has been overlooked and despised by several successive generations of physicians. He is fully persuaded of the truth of the following sentiment from Bichat: "Humoral medicine has been exaggerated without doubt; but it has solid foundations, and in a number of cases we cannot deny that all should be referred to a vice of the humors." His object, therefore, is to remove the prejudice so long and generally entertained against the application of chemistry to pathological facts, and to bring back the profession to the chemical study of the blood. He insists that it would afford proof of a culpable ignorance, or at least of a dangerous scepticism, to refuse to accept the results of the chemistry of the present day, "because those yielded by another, which had nothing in common with it but the name, had been convicted of insufficiency or of error."

We fully agree with our author, and are ready to join with him in giving thanks to organic chemistry for the aid it is bringing by its experimental methods to the science of pathology. We believe we could not bring before our readers a work abounding more in novel and striking details than this volume on hæmatology; and we therefore propose to give as full an account of its contents as our limits will allow.

The author sets out with the statement, founded on a series of experiments made by himself, Gavarret, and Delafond, that the healthy appearance of the blood is not the same in different classes of animals, and that a feature which belongs to it physiologically in other beings, is the certain expression of a morbid condition in man. For example, the buffed appearance which, in the human species, is always indicative of inflammation, is uniformly present in the blood of horses, provided the bleeding be effected in a particular way. The composition of the blood likewise varies in different classes of animals. That of the dog contains much less fibrine than that of man and of all other animals, but it is at the same time richer in globules, while, on the other hand, the horse, the sheep, and the ox, whose blood contains more fibrine than that of man, and especially than that of the dog, have in their blood decidedly a smaller proportion of globules than those races. In different individuals of the same species the several elements of the blood may present variations within certain limits without a disturbance of health. But for each of the elements there exists a maximum and minimum which cannot be passed in the physiological condition of the system. Thus, the average of the fibrine, in healthy human blood, is $\frac{3}{1000}$. It may vibrate about this average, falling to 2.5, or rising to 3.5 without affecting the physiological state. Some persons, says our author, without being sick, have nearly four in fibrin, and in others it may be as low as two; but these he regards as exceptional proportions, belonging rather to true idiosyncrasies, than to the system as ordinarily constituted.

For the globules, $\frac{127}{1000}$ is taken as the average in human blood; and this may vary from 140 to 110, which are the extreme limits compatible with health. But the maxiumum, 140, is linked with the plethoric condition, which by development becomes morbid; and the minimum is associated with a debility, congenital or acquired, which is often the beginning of disease.

The albumen is stated at $\frac{80}{1000}$ as the mean, which may be exceeded or depressed to a limited extent, in the healthy system, but if the diminution be carried to a certain degree, disease is the inevitable result.

Having determined the variations of quantity that the different elements of the blood may present in a state of health, we are prepared for the examination of that fluid in a pathological condition. This is conducted by means of the microscope and of chemical analysis. The first discloses differences in the volume or in the form of the globules, the existence of pus in the blood, and whatever modifications may occur in the size and other physical properties of the normal principles of that fluid. By the second, we arrive at a knowledge of the healthy constitution of the blood, and its various departures from a physiological state, whether from increase or diminution of its normal elements, or the formation of new principles in it.

The author begins by showing what are the changes in the blood in those two opposite states of the system—*plethora and anæmia*—which, though coincident with health, if not excessive, cannot go beyond a certain limit without inducing disease. With respect to the first condition, what does analysis teach? Our author answers, that it has shown him, in the first place, that the blood in plethora does not contain more fibrine than in any other condition, nor even tend to mount towards the higher limit of the physiological state; and, in the second place, that the albumen does not offer any remarkable change of proportions. Only the globules then remain, 'and it is effectively the great elevation of their proportion,' according to M. Andral, 'which establishes in the blood the character of plethora.' In 31 bleedings where the individuals were plethoric, he 'found for the mean of the globules, the cipher 141; for minimum, 131; and for maximum, 154.' The blood of such people then differs from ordinary blood in the excess of globules, and the much less quantity of water which it contains. Persons of this habit are subject to certain symptoms, to wit: vertigo, dizziness, heat of the head, and tinnitus aurium; but, very singularly, analogous symptoms present themselves if too small a number of globules traverse the vessels of the brain. Such subjects are not more liable to inflammations than those of a different habit; but they are more apt to suffer from hemorrhages, and the state often leads to true fever. Inflammation, moreover, occurring in such a constitution will be marked by symptoms of intense reaction.

*Of the Blood in Anæmia.*—As the characteristic feature of plethora is increase in the proportion of blood globules, so anæmia is characterised by deficiency of this element of the blood. Our author in sixteen cases of commencing anæmia found 109 to be the average proportion of the globules, and in 24 cases of confirmed anæmia he found the cypher as low as 65. These were cases of spontaneous anæmia, and he mentions that in one case of this kind he found the proportion of globules as low as 28. It is the globules alone which are diminished in spontaneous anæmia, whether the case be strongly marked or not. But if that state be induced by excessive losses of blood, the fibrine and albumen will also be diminished after a time. Thus M. Andral, in a woman who had lost much blood from metorrhagia, found the globules as low as 28, the fibrine 1.8, and the solid matter of the serum 61—the water having risen to the enormous proportion of 915.

Anæmia is occasionally an attendant upon pregnancy, in which case, as in other instances where it is spontaneous, it is only the globules which are diminished.

The long continued action of lead upon the human constitution is also known to produce this state, in which the deficiency is found to be confined to the same element of the blood. It is the belief of our author that the globules in anæmia are not only diminished in quantity, but also altered in their structure, tending to a true destruction. In two cases of chlorosis they appeared to him smaller than natural, and some had no longer their accustomed form—they appeared in the field of the microscope broken, and disseminated like kinds of fragments. The blood of anæmic individuals presents on coagulating a small clot, swimming in an abundant, colorless serosity. This clot is dense and cohesive, and not unfrequently covered by a buff, such as one would expect to find on the blood of a patient affected with pleurisy or articular rheumatism. The density of the clot, and the buff which covers it are the more marked in proportion as the anæmia is more decided. These phenomena which have long been known to the profession are thus satisfactorily accounted for by M. Andral:

I regard as incontestable, the fact that the clot of the blood of chlorotic patients is often buffed, and like Borsieri, I have from this long since drawn the inference that the presence of the buff is not always evidence of the existence of an inflammatory disease, for chlorosis is certainly not of this character. But why is the blood, in this disease, often cupped? It is because the blood of chlorotic patients has retained all its fibrine, and lost some of its globules; it is in consequence of this that there is really in this blood, as in that of inflammations, or as exists normally in the blood of some animals, excess of fibrine in proportion to the globules; now, whenever this excess takes place, whether it be absolute or relative, and whenever at the same time the coagulation of the fibrine is not very much too rapid, this principle will be seen to accumulate alone on the surface of the clot, and the cup will appear. This is the reason why the blood of anæmic individuals may by buffed, and why that of plethoric individuals is not; it is the cause, also, why the coagulum of blood is firmer and more dense in the first than in the last. It is also one of the circumstances which explains the constant existence of the buff in the blood of the horse. We must add to this, in the latter animal, the greater slowness of the coagulation of the fibrine."

The effects of the deprivation of the blood of this essential principle are given in the following passage:

"The blood cannot be deprived of a certain quantity of its globules, without there resulting from it great prostration of the muscular system, very marked general feebleness, grave perturbations of the nervous system, which are betrayed by different disorders of the intelligence, of sensation and of motion; and various disorders of the functions of digestion, respiration, and circulation. Who is ignorant of the various neuroses to which anæmic patients are subject, their dyspepsia, their dyspnœa, their palpitations of the heart? Who has not observed the singular discoloration of their skin and exterior mucous membranes, which is so naturally explained by the small number of coloring globules which still flow in their vessels? But what is less generally known is, that it is not uncommon to meet with individuals whose color remains natural, whose cheeks even are habitually injected to a remarkable extent, and whose external aspect would easily make us regard them as of a plethoric habit, but who nevertheless have in their blood an insufficient quantity of globules; this is because there exists in them a false appearance of plethora. Observe in effect such individuals a little more closely, and you will be struck with their feebleness; they will have, as in the most advanced anæmia, vertigo, dyspnœa, and palpitations, upon the slightest effort; they will bear with difficulty any kind of exertion, and still worse venesection, which far from diminishing, will increase their symptoms."

It is an interesting observation of our author, that the temperature of the human subject is not affected by the most considerable diminution of the blood globules. Thus individuals who had in their blood no more than 50, 40, 30, and even 21 parts in globules for 1,000 parts of blood, preserved nevertheless their healthy temperature. And not only so, but where these individuals are attacked with fever the heat of their bodies rises above the standard of health. It must be confessed that these facts are not very coincident with Liebig's hypothesis, which holds the globules to be carriers of oxygen.

*Of the Blood in Pyrexiæ.*—The pyrexiæ, remarks M. Andral, form a large class of acute diseases which it has been vainly sought to dismiss from nosological systems, in order to throw them all into the group of simple inflammations. He adds, that such pretensions cannot be maintained—the pyrexæ exist as diseases apart. This, he insists, is evident from the causes which develop and the symptoms which characterize them, as well as the alterations in the solids which are often posterior to the febrile movement. But it is especially by the analysis of the blood that the distinction, in his view, is made clear and conclusive. The results furnished by this analysis, he observes, have something so marked, that they seem to him to fix in a definite manner the distinction, vainly combated, between the pyrexiæ and the phlegmasiæ. In the former we find no constant alterations either in the solids or the blood that can account for the phenomena. In the latter we shall presently see the case is different. In the pyrexiæ, as our author remarks, the only phenomenon which never fails, is the fever itself. In them the fibrine never augments, supposing them free from all inflammatory complications, while in some forms of fever it is diminished to a degree not known in any other acute disease. Such fevers are termed adynamic, putrid or typhus. In them the blood seems tending towards a sort of dissolution. Whatever be the form of fever, the serum and clot are imperfectly separated; the clot is voluminous, is never elevated upon its borders, as in the phlegmasiæ, is of slight consistence, and easily broken and torn. In the adynamic form, it may often by the slightest pressure be reduced to a true condition of diffluence, in which it ceases to form a single mass, and is divided into a number of grumous portions which mix with the serum, and color it of a more or less deep red. In the early stage of these fevers the proportion of globules is often increased. M. Andral was formerly disposed to regard this high cipher of the globules as characteristic of the blood in the pyrexiæ, but he recants this opinion in the work before us. He continues his history of the blood of febrile patients in the following passages:

"There is another quite negative character of the blood in the pyrexiæ, which is of importance, and which serves still more to distinguish the sanguine fluid of these diseases from that of the phlegmasiæ. This character is the absence of the buff. I may affirm here that I have never met with it, unless there was some phlegmasial complication, either in inflammatory fever, in slight or severe typhoid fever, in measles, in scarlatina, or in variola."

"Since the diminution of the fibrine does not exist necessarily in any pyrexia, it is perfectly clear that it is not in this alteration of the blood, that we should place the point of departure of this class of diseases. But what seems to me incontestible, is, that the specific cause which gives them birth, acts upon the blood in such a way that it tends to destroy its spontaneous coagulable matter, while the cause which produces the phlegmasiæ tends on the contrary to create in that fluid a fresh proportion of that matter. If this cause act with slight energy, or if the economy resist it, the destruction of the fibrine is not accomplished; if, on the contrary, the cause continue to act with all its intensity, and the forces of the organism be in fault, the destruction of the fibrine will commence either at the very beginning of the disease, which is very rare, or a certain period after its commencement: all this applies itself equally well both to typhoid fever, and to the eruptive fevers. For me there is, in all these cases, a true intoxication; if it be slight, its effect must to be sure always exist, but it is not appreciable; if the intoxication be stronger, the effect which it has produced upon the blood becomes visible, and is marked in that fluid, by a diminution of the fibrine."

In this state of the blood we have a satisfactory explanation of the frequent occurrence of hemorrhages which has been remarked in protracted fevers, and especially in those of an adynamic type. The same condition of that fluid is found to exist in those cases of scarlatina, in which there are abundant losses of blood from all parts, and also in persons attacked with acute purpura hemorrhagica. Of the latter affection a striking case occurred in our practice a little more than a year ago. A young woman, 20 years of age, whose health had been pretty good, though not robust, found herself unusually indisposed about her monthly period. The menstrual discharge being too profuse and continuing beyond the ordinary time, her health failed her, and she was obliged to take to her bed. On the 8th day of the uterine hemorrhage her friends were alarmed by the appearance of a new symptom—she began to discharge blood from the mouth, which on examination was found to proceed from the gums. The blood was of a dark color, and formed a soft coagulum. On the next day we saw her, and found her with a rapid, soft pulse, offensive breath, anxious expression of countenane, constipated bowels, and complaining of precordial distress. The metorrhagia continued, and she was losing blood from her gums at the rate of 12 ounces in twenty-four hours. Three days after we first visited her, and on the twelfth of the disease, she died, petechiæ having appeared on her body and extremities, and her skin, especially the face, having assumed a livid hue. The treatment consisted of purgatives, quinine and stimulants, with remedies, general and local, addressed to the hemorrhage. No benefit appeared to be derived from any of them. The uterine hemorrhage rather augmented during the last days of her illness, and no astringent checked the bleeding from the gums.

M. Andral did not find that the animal temperature was lowered by a loss of a portion of the fibrine of the blood, any more than by a diminution of the globules. It is even much augmented in fevers where occasionally the blood offers not more than $\frac{1}{1000}$ in fibrine. In intermittent fevers the human temperature, according to this author, attains its maximum, and here the fibrine of the blood remains in a normal quantity.

*Of the Blood in the Phlegmasiæ.*—Having shown that in fevers the fibrine may be natural in quantity, or diminished, but never increased, our author proceeds to state, that in cases of inflammation this principle is invariably found augmented. This characteristic is never absent in the blood of persons afflicted with inflammatory diseases. The words of M. Andral are:

"In the phlegmasiæ, then, there is an excess of fibrine relatively to the globules, that is to say the reverse of what takes place in typhus. Hence may be explained the physical properties of the blood drawn from a vein, in these diseases. While in the pyrexiæ, generally, the clot is bulky, flabby, and imperfectly separated from the serum, here, on the contrary, it is smaller, more dense, and of superior consistence; besides which, if the blood has been properly drawn, the clot will be covered with a buffy coat of variable thickness.

"I have already explained myself in regard to the value of the indications to be derived from the buffy coat. Except when it occurs in cases of anæmia, this production uniformly denotes a state of inflammation. I can cite in support of this assertion a summary of nearly eighteen hundred bleedings, in which the blood, examined by myself, never presented a buffy coat except in one or the other of two series of cases, those of anæmic patients on the one hand, and those of persons attacked with various acute or subacute phlegmasiæ, on the other. In the former, the buffy coat, which, indeed, is of moderate thickness, results from the great diminution of the globules, for the fibrine, although in its usual quantity, is nevertheless in excess, relatively to the globules. But in the phlegmasiæ the globules are neither increased nor diminished, while the fibrine having become redundant, the law which governs the formation of the buffy coat reaches its full development. Moreover, the fibrine of the new product entering into the composition of the buffy coat coagulates more slowly than the old, which is another cause favorable to the appearance of the buffy coat, since the gradual coagulation of the fibrine allows the globules to sink to the bottom of the vessel, leaving the fibrine above them, still dissolved, or suspended, in the serum."

Here then is the essential modification of the blood in inflammation—the creation of a new quantity of fibrine. In this consists the great difference of the blood in fevers and the phlegmasiæ. It presents itself if inflammation be developed in the progress of typhoid fever. But in this case it never attains to a high degree. Even in anæmic subjects inflammation is attended by this modification of the blood. So the conclusion is, that be the state of the system what it may, the supervention of an acute phlegmasia involves necessarily, and in every case, the increase of fibrine in the blood beyond its normal quantity. Our author continues:

"In man, when an acute inflammation is well established, the fibrine varies in quantity between 6 and 8; in a smaller number of cases it rises as high as between 8 and 9; and, more rarely still, exceeds the latter number, and reaches by degrees 10½, which is the highest number I have yet found representing the fibrine in phlegmasiæ attacking the human race, though in a neat affected with pneumonia I found it as high as 13. It must be remarked here, that since the physiological number representing the fibrine in animals is not the same as that for man, the increment in the former must not be judged of by the standards of the latter, but must be referred to the average obtained for each particular class of animals. Thus the physiological mean of the fibrine in dog's blood being only 2.1, the numbers 3, 4, and 5 obtained when this animal was attacked with inflammation, would, in the case of man, indicate a development of fibrine to be represented by much higher numbers. On the other hand, the physiological mean of the fibrine in the blood of horses and neat-cattle being greater than that of man, the number 13 indicating the quantity of fibrine in the blood of one of these animals, would, in the case of man, represent a much inferior quantity. These corrections are indispensable, if we desire profitably to apply to human pathology the results obtained from studying the pathology of other species."

The greatest development is in pneumonia and acute articular rheumatism. In them the augmentation has reached as high as 10½—more than three times the natural quantity. It is less in inflammations of the mucous surfaces. In mercurial stomatitis it is increased, and is shown by cases cited by our author to be augmented in proportion to the extent and intensity of the inflammation. In one case of severe salivation he found the fibrine had risen to 6.6.

From this it is plain that we have erred in ascribing to mercury any power of dissolving the blood. On this point M. A. expresses himself to the following effect:

"Mercurial stomatitis, therefore, notwithstanding its specific nature, does not differ from ordinary inflammations in its influence on the blood; and yet it has been asserted again and again, that one of the effects of mercury introduced into the system is to bring about a state of dissolution of the blood, which is incompatible with an increase of fibrine. It is possible that this may take place after a prolonged use of the medicine, but assuredly such is not the case soon after its first exhibition. Consequently, when it is administered to combat certain acute phlegmasiæ, such as peritonitis, it is not right to assume that its antiphlogistic action consists in its producing in the blood a condition opposed to that which belongs to the inflammatory state. Nor do I find that this dissolving influence upon the blood which is claimed for mercury, has ever been demonstrated, in any alleged case, by a rigorous examination of that fluid. It appears to me that the opinion rests chiefly on a fancied analogy between the effects of mercury, and those of scurvy, upon the mouth."

We published the history of an interesting case of purpura hemorrhagica in the first volume of the Transylvania Journal of Medicine, February, 1828, and in looking around for the exciting cause of the disease, we hinted that it might have been excited by the use of calomel, which had been given by our directions to the extent of producing slight ptyalism. We were most reluctant, at the time, to admit the agency of the mercurial in provoking the hemorrhage, and are now fully convinced by the testimony of M. Andral, that it was quite guiltless in the case.

Having cited many special inflammations in which the increase of fibrine is conspicuous, our author continues:

"The increase of fibrine in the blood takes place from the very commencement of the inflammatory state. I have frequently had the same individual bled twice; the first time, on the day previous to the inflammatory attack, and the second time, a few hours after its distinctly marked invasion. The first time, the fibrine of the blood was normal, the second, in excess. I have, thus far, vainly striven to determine whether or not the composition of the blood was modified, before the change denoting inflammation appeared in the solids; I have not succeeded, and my analyses have proved to me nothing more than the simultaneous origin of these two phenomena."

This change in the composition of the blood must in some instances be subsequent to alterations in the solids. Such is the inflammation from burns, where the solids are first to suffer,but where the blood is found to have undergone this modification. On the contrary, in articular rheumatism the change in the solids is slight, while the increase of fibrine is very great. Inflammation, our author hence concludes, is not a disease seated in the solids merely, nor yet a purely local affair confined to the solids.

Before dismissing this subject, the author speaks of the remedies by which excess of fibrine in the blood is corrected; and first of bloodletting.

"Amongst the various forms of this treatment depletion holds the first rank. I have then naturally to inquire how far bleeding, repeated more or less frequently, has the power of removing the excess of fibrine in the blood, rapidly or gradually. Now it is found that however repeated or abundant the bleedings, the fibrine increases none the less, if these bleedings are performed in the early stages of an inflammation of some intensity, or, in other words, at the period of the ordinary increase of the disease; on the other hand the inflammation does not prevent there being found, after each bleeding, a progressive diminution of the globules. It seems, then, that when once the blood has set about producing an excess of fibrine, no matter what is done, a certain time must elapse before this disposition is exhausted. Besides, this resistance of the fibrine to the action of depletions, and the development it acquires in spite of them, are perfectly in keeping with what takes place in the inflamed solid itself, and in the rest of the organism. The most copious loss of blood does not effect the immediate removal of the lesions of the solid; a certain space of time is always necessary for accomplishing this, and for the extinction of the fever. So that the fibrine, the quantity of which in the blood represents the degree of inflammation, obeys the same law which makes the latter continue for a certain time, and pass through certain stages. Let it not, however, be thought that I deny the utility of blood-letting, when properly employed, in this class of diseases. Experience has taught me, that without removing them suddenly, it often abridges their duration, and conspires to bring about a favorable issue. I even admit that if blood be drawn at the very outset of the inflammation, while yet there is nothing more than congestion in the solid, and the fibrine of the blood hardly above its normal standard, depletion may stop the progress of the disease, and, in certain cases at least, render it really abortive. But if the disease be a little farther advanced, this will not be the case: it is not in the power of art to prevent a well-formed pneumonia from lasting seven or eight days at least, although it may prevent its being prolonged for a fortnight. You cannot arrest a well marked case of acute articular rheumatism within eight, twelve, and oftener, fifteen or twenty days; but by the use of blood-letting will more frequently see it arrested within the last named period, than if depletion had not been used."

*Of the Blood in Hemorrhages.*—Some of these are connected with no appreciable lesion of the solids, and must, therefore, have their origin in a change in the composition of the blood. This consists in a relative or absolute diminution of its fibrine. We see it in scurvy, purpura, typhus fever, &c. It is the reverse of what occurs in the phlegmasiæ.

"Hemorrhagic blood, as regards its physical properties, does not differ from that of the pyrexiæ. It never presents any buffy coat, without inflammatory complication. The clot is generally large, and never small, except in those cases of extreme poverty of the blood of which I spoke last. It is more commonly remarkable for considerable softness, and when the hemorrhage depends on a very great diminution of fibrine, the blood may be so little coagulable as hardly to form a veritable clot; or it may happen that, instead of this latter, there is nothing in the vessel containing the blood, except some disconnected lumps suspended in reddish serum. This is that state of dissolution of the blood of which I have already spoken under the head of the pyrexiæ, and which exists, more or less, in all the diseases characterized by a general disposition to hemorrhage."

The alkalis produce this effect on the blood. Magendie threw into the veins of a living animal a concentrated solution of the carbonate of soda, and found that an almost fluid blood was the result, and that the symptoms during life were nearly the same as those observed in diseases in which the older writers admitted a state of dissolution of the blood. It has been declared by some authors that they have found an excess of alkali in the blood of persons who died of low fevers or of scurvy. Our author supposes the venom of the viper and various miasmatic substances may act by inducing this state of the blood.

It is worthy of remark that this dissolved character of the blood, if we may judge from the histories of epidemics by writers before the eighteenth century, was a much more common thing in ages past than it is at the present day. Those authors continually speak of dissolved and incoagulable blood, as a characteristic of the fevers of their times. Huxham, as quoted by Andral, describes very forcibly these appearances. In his narrative of the epidemic fever of Plymouth, he mentions that the blood did not present even a distinct coagulum, but the vessel appeared to contain simply a brown liquid, at the bottom of which was deposited a sort of reddish powder, formed by the separate particles of what should have been the clot. One cannot help being struck with the coincidence between this condition of the blood, and the hygienic circumstances which were in operation at the time. We find Erasmus, for example, writing towards the close of the sixteenth century, that "the inhabitants of London were every year, from spring to harvest, attacked by a malignant fever, which committed the greatest ravages in that city, and especially amongst the poorer classes."

"The supply of water," he adds, "fails the inhabitants; they have to seek it at a great distance from the city; the river water is carried upon their backs, and is so dear that the poor cannot procure enough of it to wash themselves, and keep their houses clean. These houses are of wood, and very cold in winter, which makes it necessary to fill the rooms with straw. But as this cannot be often renewed, it becomes spoiled, and very injurious."

A striking case illustrative of what the old writers considered "a dissolved and putrid state of the blood," as quoted by our author from Huxham's Essay on Fevers. The subject of it was seized with alternate rigors and flushes of heat, his pulse was weak, and he had general debility. Four or five days after this, his breath was observed to be fetid; he was still going about, but suddenly, two days after, he fainted. It was found that his neck and hands had livid spots on them. He fainted repeatedly, his breath had become insupportably fetid, blood leaked from his gums, and a great many livid, violet and black spots appeared all over his body, on the trunk as well as the limbs. A violent hemorrhage broke forth from his nose, and blood dropped from the caruncle of one of his eyes; several livid pustules appeared on his tongue, and the inner side of his lips, discharging a thin bloody matter when they broke. A bloody dysentery came on—his pulse intermitted every sixth or eighth pulsation—the dysentery being restrained, his urine became tinged with blood. The blood drawn from him seemed a '*purulent sanies*' rather than blood, with a kind of sooty powder at bottom. Still, the patient recovered on a course of bark, with aromatic and astringent substances.

Maladies characterized by these symptoms now rarely appear, especially of the hemorrhagic type, and never except in sporadic cases, and scurvy has also ceased as an endemic disease.

*Of the Blood in Dropsies.*—While some forms of dropsy arise from disease of the solids, it is generally acknowledged, says, M. A., "that there are others again whose point of departure must be sought for in an alteration of the blood." This has been said to consist in an impoverishment of that fluid. What is meant by that expression? The blood may be degenerated by a loss of fibrine, globules, or albumen. In all such cases, that fluid may be said to be impoverished. Is dropsy equally the result of each condition? We have seen that diminution of fibrine does not lead to it, and our author correctly objects to its being a result of the loss of the globules ,that it is not common in chlorotic cases, nor in cases of spontaneous anæmia in men. It is diminution of the albumen, then, which results in the production of dropsy. And here we have an explanation of the fact that in dropsy the urine is so often albuminous. Such is the character of that secretion whether the dropsy ensue upon scarlatina, exposure to cold, or insufficient alimentation. Sheep, upon a bad diet, and in low situations, become dropsical, with blood deficient in albumen.

*Of the Blood in Organic Diseases.*—In five cases of hypertrophy of the heart, M. Andral found the albumen and globules normal—the fibrine in one was as high as 4. In this there was a rheumatic complication. No increase of this principle is found unless inflammation be present. It is not augmented by the deposit of tubercles, encephaloid, or scirrhous matter, a proof that in their first stage these productions do not lead to inflammation. "So long," remarks our author, "as tubercle and cancer preserve the character of hard masses, without any inflammation around them, an analysis of the blood uniformly gives the normal quantity of fibrine." This begins to increase only when the process of softening is set up, and inflammation is established. We have the proof of the fact in the following quotation:

"We weighed the fibrine obtained from the blood of thirty tuberculous patients, drawn at thirty-three different bleedings. In seven of these patients the tubercles were still crude; in nine others they were softening, and in the remaining fourteen, there were cavities in the lungs.

"The six patients of the first series were bled, altogether, nine times. Seven times the fibrine was found to be normal, varying between 2.7 and 3.5. Twice, however, the fibrine exceeded its physiological quantity, and gave the numbers 4.8 and 5.1. But, in each of these two cases, there was an inflammatory complication; in one a sub-acute entero-colitis, and, in the other, a bronchitis of much greater intensity than is usual in the first stage of phthisis.

"In ten bleedings performed on the nine patients of the second series, we already come to different results. Nine times out of ten the fibrine was in excess, sometimes very slightly, hardly reaching 4, and sometimes varying between 4 and 5. In the tenth case, there were only three parts of fibrine.

"In fourteen bleedings performed on the fourteen patients of the third series, the fibrine was in excess twelve times, but much more so than in the second series. The minimum found was 4.0, and that once only. In three other cases, the fibrine varied between 4.4 and 4.6; in all the others it was between 5.0 and 5.9.

"The only two cases of the third series which did not follow the rule of the increase of fibrine, must be regarded as exceptional; the subjects of them were far gone in marasmus, when we attempted, by a small bleeding, to diminish the state of half asphyxia which they presented; and, indeed, they were momentarily relieved by it. In one of them the fibrine was at its physiological mean, in the other it had fallen to 2.0.

"In the third stage of phthisis, therefore, although the fibrine is generally in excess, there are yet some cases, in which the exhaustion, consequent on the softening of the tubercles, is indicated by an opposite modification in the amount of fibrine; it augments during the process of elimination, and then diminishes, so as to fall even below the lowest limit of its physiological state."

We had wished to notice other parts of this original and instructive Essay, but find our limits are exhausted. We commend the subject of which it treats so ably, to the attention of all who are ambitious of keeping up with the progress of pathological science in this interesting quarter. Y.

